# The prevalence and correlates of substance use disorders among patients of two different treatment settings in Thailand

**DOI:** 10.1186/s13011-021-00345-2

**Published:** 2021-01-13

**Authors:** Supa Pengpid, Karl Peltzer

**Affiliations:** 1 ASEAN Institute for Health Development, Mahidol University, Salaya, Phutthamonthon, Nakhon Pathom, Thailand; 2 Department of Research Administration and Development, University of Limpopo, Turfloop, South Africa; 3 Department of Psychology, University of the Free State, Bloemfontein, South Africa

**Keywords:** Substance use disorders, Different treatment settings, Thailand

## Abstract

**Background:**

Monk healers provide an accessible and popular service in Southeast Asia, but little is known on the substance use status of their clients. This investigation intended to assess and compare the rate and correlates of substance use disorders in two different treatment settings (monk healers = MH and primary health care = PHC) in Thailand.

**Methods:**

In a cross-sectional study, 1024 patients (591 of MH and 613 of PHC) responded to screening measures of the “World Health Organization Alcohol, Smoking, and Substance Involvement Screening Test Lite”, and two common mental disorders (major depression and generalized anxiety disorder) from November 2018 to February 2019. Logistic regression was used to estimate the determinants of any substance use disorder in the MH and PHC setting.

**Results:**

The prevalence of substance use disorder was higher in MH clients than PHC patients: any substance use disorder 11.7% (95% Confidence Interval-CI: 9.3–14.5%) vs 5.4% (95% CI: 3.9–7.5%), tobacco use disorder 7.6% (95% CI: 5.7–9.9%) vs 2.5% (95% CI: 1.5–4.0%), alcohol use disorder 10.0% (95% CI: 8.4–13.6%) vs 4.3% (95% CI: 3.0–6.3%), any drug use disorder 4.2% (95% CI: 2.8–6.1%) vs 0.3% (95% CI: 0.08–1.3%), and any past three months drug use 8.2% (95% CI: 6.2–10.7%) vs 1.5, 95% CI: 0.8–2.8%). In adjusted logistic regression analysis, among MH clients, male sex (Adjusted Odds Ratio-AOR: 9.52, 95% Confidence Interval-CI: 5.06–17.92) was positively, and were married (AOR: 0.32, 95% CI: 0.16–0.61) and high social support (AOR: 0.40, 95% CI: 0.16–0.99) were negatively associated with any substance use disorder. Among PHC patients, male sex (AOR: 7.05, 95% CI: 2.99–16.63) was positively and age (AOR: 0.95, 95% CI: 0.92–0.98) was negatively associated with any substance use disorder.

**Conclusion:**

The proportion of substance use disorders among MH attendees was more than twice that of PHC attenders in Thailand, calling for collaboration in controlling substance use disorders between the two treatment systems.

## Introduction

Globally, traditional health practitioners (THP) have been shown to contribute to mental health services [1]. An earlier review described the potential benefits of traditional healing in the prevention and treatment of substance use disorders [2], and in a more recent evidence review on complementary medicine in treating substance use disorders showed that mind-body interventions, herbal therapies and acupuncture demonstrated promising results [3]. Hai et al. [4] found in a systematic review, evidence of the efficacy of spiritual/religious interventions for substance use disorders.

Monk healers (MH) or THP have been described as providing treatment for substance use disorders in Thailand [5, 6], in Malawi [7], in South Africa [8], and in the Americas [2]. “Buddhist monks who are Thai THP are called *maw pra* (monk healer), and they provide treatment, including Thai traditional medicine and indigenous practices, to the public at the Buddhist temple where they reside.” [9–12]. MH operate in various locations in Thailand [3], making use of different treatment modalities, such as herbal medicines and prayers [10–12]. For example, in the Buddhist monastery Wat Tham Krabok in central Thailand, a traditional treatment programme for substance use disorders includes Buddhist religious principles, traditional herbal medicine and physical therapy [5]. The Thamkrabok substance abuse treatment programme has been in operation since 1959, and has a monthly intake of 300–400 addicts [6].

In a study among THP attenders in Kenya, the prevalence of alcohol misuse was 9% [13]. In a national survey in South Africa, the use of THP was predicted by those who had a substance use disorder [14]. Several studies on the prevalence of common mental disorders but not substance use disorders in the THP setting were carried out in Africa [15–17]. For example in urban Tanzania, the proportion of common mental disorders of THP clients was 48 and 24% among PHC patients [16]. Based on this study, we hypothesised that the prevalence of substance use disorder would be higher in MH clients than PHC attendees.

Studies reporting the prevalence of substance use disorders in Thailand include 5.6% prevalence of any substance use disorder in Bangkok, Thailand [18], 33.0% of moderate to high-risk substance use in primary care in southern Thailand [19], and 31.1% moderate or high risk of alcohol or tobacco use among men attending district hospitals in central Thailand [20]. In other countries, the prevalence of 12-month any substance use disorder in primary care ranged from 3.2% in Spain [21] to 35.8% in USA [22]. In Nepal, one in every 14 (7.5%) primary care users had an alcohol use disorder [23]. In the USA, in urban primary care, the prevalence of moderate- to high-risk tobacco use was 15.3%; alcohol use 8.5%, cannabis use 5.1%, cocaine use 2.5% and opioid use 2.5% [24]. Correlates of substance use disorder in primary care, include male sex [23, 25], lower education [20], younger age [20], being separated, divorced or widowed [20], co-occurrence of depression or anxiety disorder [26], chronic conditions [27], not obese and not having diabetes [20].

There are no studies on the prevalence of substance use disorders in the THP setting in comparison to the PHC setting in Asia. Consequently, this study aimed to assess the rate and correlates of substance use disorders among MH or PHC attendees in Thailand.

## Methods

### Study design and participants

In all, 1024 adult MH or PHC patients (age range: 19–93 years) were consecutively recruited prior to health-care consultation (every person was eligible) into the cross-sectional study from November 2018 to February 2019; response rate 97%. Three MH and three PHC sites were purposefully sampled from two regions of Thailand. Inclusion criteria for the six sites were five or more adult attendees a day. MH and PHC attendees inclusion criteria were able to comprehend Thai language, willingness to provide informed consent, and aged 18 years and older.

Sample size calculation. Using the assumptions of the prevalence of substance use disorder of 5.6% in the health care setting (based on a previous study [18]) and a 5% higher prevalence in the MH setting, we predicted 10.6% in the MH setting (5.6 + 10.6/ 2 = 8%) cluster = 3; confidence interval = 95%, acceptable margin of error 3%, minimum cluster size =105, and minimum sample size is 315. In this study, we collected more than 590 in each setting, which is greater than the minimum sample size.

Trained professional nurses collected background data and substance use disorders in all study sites using interviews with structured questionnaires in Thai language that had previously been pretested for validity. The study protocol received approval by the “Office of The Committee for Research Ethics (Social Sciences), Mahidol University (No.: 2017/055.1403)”, and written informed consent was obtained from all participants.

### Measures

The “*Ultrarapid Alcohol, Smoking, and Substance Involvement Screening Test* (ASSIST-Lite)” optimized for general medical settings was used to assess substance use disorders [28]. ASSIST-Lite asked the past 3 months use of psychoactive substances, specifically, “smoking tobacco, alcohol, cannabis, amphetamine-type stimulants (or cocaine or a stimulant medication not as prescribed), sedatives (or sleeping medication not as prescribed), and street opioids (or an opioid containing medication not as prescribed).” [28] “For each substance there are two (or three, for alcohol) questions to determine level of use, and the ASSIST-Lite scores them as follows: Not used in the past 3months (0); l Used, but no other questions positive (1); l Used and either question positive (2); l Used and both questions positive (3). The cut-off score for a likely substance use disorder is 2, and for alcohol use disorder is 3.” [28] The ASSIST-Lite has been validated in several western and eastern cultures, including Thailand [28]. Cronbach alpha of the ASSIST-Lite was 0.90 in the current study sample.

*Major depressive disorder* was assessed with in Thailand validated “*Patient Health Questionnaire-9* (PHQ-9).” [29, 30]. Cronbach’s alpha of the PHQ-9 was 0.88 in this sample.

*General anxiety disorder* was measured with the “Generalized anxiety disorder 7-item=GAD-7).” [31]. Cronbach’s alpha of GAD-7 was 0.92 in this study sample.

*Social network support* was sourced from the “*Oslo 3-items Social Support Scale (OSSS-3)”,* covering “the number of people the respondent feels close to, the interest and concerns shown by others, and the ease of obtaining practical help from others.” [32]. Summed scores were classified into “3-8=poor, 9-11=moderate, and 12-14 strong support.” [32]. Cronbach’s alpha of the OSSS-3 was 0.75 in the current study sample.

Chronic conditions. Clients were asked about 13 health care provider’s diagnosed chronic conditions, as used in previous surveys in primary care and traditional and complementary medicine settings [33–35]. Chronic conditions included cancer or a malignancy of any kind (malignant neoplasms), diabetes or blood sugar, emphysema/asthma, heart attack or angina, high blood cholesterol, hypertension or high blood pressure, osteoporosis, sore joints, migraine headaches, ulcer (a stomach, duodenal or peptic ulcer), fatigue disorder, sleeping problem, and stroke.

*Sociodemographic information* comprised extent of debt, highest level of education, religion, employment status, age, and gender.

### Statistical analysis

“IBM-SPSS for Windows, version 25 (Chicago, IL, USA)” was used to analyse the data. Apart from descriptive statistics, Pearson chi-square and Fisher’s exact tests were applied for differences in proportions, and Student’s t test for differences in means. Univariate and multivariable logistic regression analyses were utilized to estimate the determinants of substance use disorders by type of treatment setting. Co-variates included age, sex, education, marital status, debt, social support, chronic conditions, and major depressive and/or generalized anxiety disorder. Variables significant at *p* < 0.05 in the univariate analysis formed part of the final model.

## Results

### Participants’ characteristics

The sample comprised of 1204 participants, 613 (50.9%) from PHC and 591 (49.1%) from MH. All MH and PHC attendees were Buddhists and the majority were women. MH attendees had higher education, higher social support, less debt, were younger, less likely married, and were more likely to have common mental disorders than PHC attendees (Table 1).

**Table 1:** Sociodemographic and substance use disorder characteristics of participants (*N* = 1204)

Variable	MH	PHC	P-value^a^	MH	PHC	P-value^a^
	Sample	Sample		Any substance use disorder		
	M (SD)	M (SD)		M (SD)	M (SD)	
Age (years)	47.3 (13.8)	53.3 (14.1)	< 0.001	44.3 (12.7)	47.4 (14.5)	0.296
	N (%)	N (%)		N (%)	N (%)	
All	591 (49.1)	613 (50.9)		69 (11.7)	33 (5.4)	
Sex						
Female	451 (76.3)	443 (72.3)	0.267	28 (40.6)	10 (30.3)	0.298
Male	140 (23.7)	170 (27.7)		41 (59.4)	23 (69.7)	
Formal education						
Primary or less	225 (38.5)	394 (64.5)	< 0.001	29 (43.3)	16 (48.5)	0.256
Secondary	185 (31.7)	163 (26.7)		24 (35.8)	13 (39.4)	
Post-secondary	174 (29.8)	54 (8.8)		14 (20.9)	4 (12.1)	
Marital status						
Single/divorced/separated/widowed	246 (41.7)	139 (22.7)	< 0.001	40 (58.8)	6 (18.2)	< 0.001
Married	344 (58.3)	473 (77.3)		28 (41.2)	27 (81.8)	
Employment status						
No	190 (32.4)	153 (25.1)	0.132	20 (29.4)	6 (18.2)	0.244
Yes	397 (67.6)	456 (74.9)		48 (70.6)	27 (81.8)	
In debt						
No/Little	426 (72.1)	454 (74.1)	0.522	49 (71.0)	26 (78.8)	0.405
High	165 (27.9)	159 (25.9)		20 (29.0)	7 (21.2)	
Social support						
Poor	104 (17.8)	102 (16.9)	< 0.001	17 (24.6)	3 (9.4)	0.098
Moderate	272 (46.6)	375 (62.0)		37 (53.6)	24 (75.0)	
Strong	208 (35.6)	128 (21.1)		15 (21.7)	5 (15.6)	
Chronic conditions						
None	211 (35.9)	232 (38.2)	0.711	29 (42.6)	12 (36.4)	0.832
1–2	228 (38.8)	228 (37.5)		22 (32.4)	12 (36.4)	
3 or more	148 (25.2)	148 (24.3)		17 (25.0)	9 (27.3)	
Major depression and/or generalized anxiety disorder						
No	462 (88.6)	505 (83.5)		47 (69.1)	23 (71.9)	
Yes	126 (21.4)	100 (16.5)	0.027	21 (30.9)	9 (28.1)	0.779

*MH* Monk healer setting, *PHC* Primary Health Care setting, *M* Mean, *SD* Standard deviation, ^a^Based on Chi-square or Fisher’s exact test

### Prevalence of substance use disorder

The proportion of any substance use disorder was significantly higher in MH clients (11.7, 95% Confidence Interval-CI: 9.3–14.5%) than PHC patients (5.4, 95% CI: 3.9–7.5%) (*P* < 0.001). Similarly, the proportion of each substance use disorder was significantly higher in MH clients (tobacco use disorder 7.6, 95% CI: 5.7–9.9%, alcohol use disorder 10.0, 95% CI: 8.4–13.6% any drug use disorder 4.2, 95% CI: 2.8–6.1%, and any past three months drug use 8.2, 95% CI: 6.2–10.7%) than in PHC patients (tobacco use disorder 2.5, 95% CI: 1.5–4.0%, alcohol use disorder 4.3, 95% CI: 3.0–6.3%, any drug use disorder 0.3, 95% CI: 0.08–1.3% and any past three months drug use 11.5, 95% CI: 0.8–2.8%) (Table 2).

**Table 2:** Proportion of each substance use disorder of participants (*N* = 1204)

Variable	MH		PHC		P-value^a^
	N	% (95% CI)	N	% (95% CI)	
**Any substance use disorder**	69	11.7 (9.3–14.5)	33	5.4 (3.9–7.5)	< 0.001
Tobacco use disorder	48	7.6 (5.7–9.9)	16	2.5 (1.5–4.0)	< 0.001
Alcohol use disorder	64	10.0 (8.4–13.6)	28	4.3 (3.0–6.3)	< 0.001
**Any drug use disorder**	25	4.2 (2.8–6.1)	2	0.3 (0.08–1.3)	< 0.001
Cannabis use disorder	8	1.3 (0.7–2.6)	1	0.2 (0.02–1.1)	0.015
Amphetamine use disorder	22	3.6 (2.4–5.3)	0	0.0	< 0.001
Sedative use disorder	14	2.2 (1.3–3.7)	4	0.6 (0.2–1.6)	0.014
Opioid use disorder	1	0.2 (0.02–0.1)	0	0.0	0.490
Past three months any drug use	49	8.2 (6.2–10.7)	9	1.5 (0.8–2.8)	< 0.001

*MH* Monk healer setting, *PHC* Primary Health Care setting, ^a^Based on Chi-square or Fisher’s exact test

### Univariate association with any substance use disorder

In the univariate logistic regression analysis, among MH clients, male sex was positively, and age, being widowed, divorced, or single and high social network support were negatively associated with any substance use disorder. Among PHC patients, male sex was positively and age was negatively associated with any substance use disorder (Table 3).

**Table 3:** Univariate associations with any substance use disorder by treatment setting

Variable	MH		PHC	
	COR (95% CI)	p-value	COR (95% CI)	p-value
Age in years	0.98 (0.96, 0.99)	0.040	0.97 (0.94, 0.99)	0.019
Sex				
Female	1 (Reference)		1 (Reference)	
Male	8.00 (4.51, 14.18)	< 0.001	6.25 (2.79, 13.99)	< 0.001
Formal education				
Primary or less	1 (Reference)		1 (Reference)	
Secondary	1.15 (0.62, 2.15)	0.663	2.04 (0.93, 4.47)	0.077
Post-secondary	0.66 (0.33, 1.34)	0.247	1.33 (0.37, 4.75)	0.666
Marital status				
Single/divorced/separated/widowed	1 (Reference)		1 (Reference)	
Married	0.45 (0.26, 0.78)	0.004	1.26 (0.50, 3.15)	0.627
Employment status				
No	1 (Reference)		1 (Reference)	
Yes	1.13 (0.63, 2.04)	0.674	1.73 (0.65, 4.62)	0.273
In debt				
No/Little	1 (Reference)		1 (Reference)	
High	1.24 (0.71, 2.17)	0.443	0.82 (0.35, 1.92)	0.640
Social support				
Poor	1 (Reference)		1 (Reference)	
Moderate	0.72 (0.39, 1.35)	0.310	2.42 (0.71, 8.19)	0.156
Strong	0.36 (0.17, 0.76)	0.008	1.64 (0.38, 7.02)	0.589
Chronic conditions				
None	1 (Reference)		1 (Reference)	
1–2	0.78 (0.43, 1.41)	0.416	1.43 (0.63, 3.25)	0.393
3 or more	0.87 (0.46, 1.85)	0.676	1.95 (0.80, 4.77)	0.143
Major depression and/or generalized anxiety disorder				
No	1 (Reference)		1 (Reference)	
Yes	1.71 (0.98, 2.99)	0.058	2.10 (0.94, 4.68)	0.070

*MH* Monk healer setting, *PHC* Primary Health Care setting, *COR* Crude odds ratio, *CI* Confidence Interval

### Multivariable association with any substance use disorder

In adjusted logistic regression analysis, among MH clients, male sex (Adjusted Odds Ratio-AOR: 9.52, 95% Confidence Interval-CI: 5.06–17.92) was positively, and were married (AOR: 0.32, 95% CI: 0.16–0.61) and high social support (AOR: 0.40, 95% CI: 0.16–0.99) were negatively associated with any substance use disorder. Among PHC patients, male sex (AOR: 7.05, 95% CI: 2.99–16.63) was positively and age (AOR: 0.95, 95% CI: 0.92–0.98) was negatively associated with any substance use disorder (Table 4).

**Table 4:** Multivariable associations with any substance use disorder by treatment setting

Variable	MH		PHC	
	AOR (95% CI)^a^	p-value	AOR (95% CI)^a^	p-value
Age in years	1.00 (0.98, 1.02)	0.698	0.95 (0.92, 0.98)	< 0.001
Sex				
Female	1 (Reference)		1 (Reference)	
Male	9.52 (5.06, 17.92)	< 0.001	7.05 (2.99, 16.63)	< 0.001
Marital status				
Single/divorced/separated/widowed	1 (Reference)		–	
Married	0.32 (0.16, 0.61)	< 0.001		
Social support				
Poor	1 (Reference)		–	
Moderate	1.00 (0.47, 2.12)	0.999		
Strong	0.40 (0.16, 0.99)	0.048		

*MH* Monk healer setting, *PHC* Primary Health Care setting, *AOR* Adjusted odds ratio, *CI* Confidence Interval, ^a^Adjusted for all variables in the Table

## Discussion

The study found that the prevalence of any substance use disorder among MH attenders was more than twice that of PHC attenders, confirming our study hypothesis. The prevalence of alcohol use disorder in the MH setting (10.0%) was similar to what was found among THP attendees in Kenya (9%) [13]. Prevalence of any substance use disorder in the PHC setting in this study (5.4%) was similar to a community-based study in Bangkok, Thailand (5.6%) [18], was higher than among PHC users in Spain (3.2%) [21], but seems lower than what was found in a study among health care attendees in Southern Thailand (33.0% moderate-high risk substance use) [19], among male out-patients in central Thailand (31.1% moderate or high risk alcohol or tobacco use) [20], and PHC patients in USA (35.8%) [22]. The higher prevalence of substance use problems in the study in southern Thailand may be explained by the selection of the health care sites (district hospital and primary care centres) in areas known to have high substance use problems [19], and in the study in central Thailand only male patients attending district hospitals were included [20], with men expected to have a higher prevalence of substance use disorder than women. The prevalence of alcohol use disorder in the PHC setting in this study (4.3%) was lower than in a study among PHC users in Nepal (7.5%) [23], and in USA (8.5%) [24]. The two major substances abused in this study were alcohol and tobacco, followed by other drugs. Similar results were found in previous studies in the PHC setting in Thailand [19], in South Africa [25], and in USA [24].

In agreement with former research [20, 23, 25, 36, 37], this survey showed that male sex, younger age, lack of social support, and being single, separated, divorced, or widowed were associated with substance use disorders in the MH and/or PHC setting. Sex specific role expectations and norms, such as associating substance use with masculinity, may be related to the male preponderance of any substance use disorder [38, 39]. Reduction in the prevalence of substance use disorders with age is expected, due to a decreased tolerance towards substances with ageing [40, 41]. Perceived social support, including being married, has been shown to be protective against substance use disorder [36].

A previous study in Thailand found an association between having primary or less education and substance use disorder [20], while this study did not find such an association. This study also did not find an association between economic status (extent of debt) and substance use disorder, unlike in some previous research [42]. While a previous study [27] found that the prevalence of substance use disorders increased with multiple chronic conditions, this study did not find this association. Consistent with a previous investigation [26], this study found that having depression or anxiety was in univariate analysis marginally associated with a substance use disorder.

### Implications

Considering the high prevalence of any substance use disorder in the MH setting, it appears that MHs are preferred over PHC centres, which provide “preventive, promotive and basic curative care” [43], in dealing with substance use disorders in Thailand. More studies are called for on the treatment modalities of substance use disorders by MHs. This may include naturalistic prospective investigations on the treatment outcome of substance use orders [16]. To reduce the gap in the treatment of substance use disorders, especially in rural communities in Thailand, MHs should be trained in evidence-based treatment practices [17].

#### Study limitations

Substance use disorders were only assessed with the use of a brief screening measure. Data were assessed based on self-reported answers. However, a large number of studies about substance use disorders are based on self-reported data. Future studies should include structured psychiatric assessments. Furthermore, the selection of the sites was purposefully, which is an additional study limitation.

## Conclusion

The proportion of substance use disorders among MH attendees was more than twice that of PHC attenders in Thailand, calling for collaboration in controlling substance use disorders between the two treatment systems. Monk healers should be trained in evidence-based practices to reduce substance abuse. Prospective investigations are needed on the treatment approach of substance use disorders by monk healers.

## Data Availability

The dataset used and/or analysed during the current study are available from the corresponding author on reasonable request.

## Abbreviations

ASSIST-Lite: Ultrarapid Alcohol, Smoking, and Substance Involvement Screening Test
GAD: Generalized anxiety disorder
MDD: Major depressive disorder
MH: Monk healer
PHC: Primary Health Care
OSSS-3: Oslo 3-items Social Support Scale
PHQ: Patient Health Questionnaire
THP: Traditional Health Practitioner

## References

[1] Nortje G, Oladeji B, Gureje O, Seedat S (2016). Effectiveness of traditional healers in treating mental disorders: a systematic review. Lancet Psychiatry.

[2] Jilek WG (1994). Traditional healing in the prevention and treatment of alcohol and drug abuse. Transcult Psychiatric Res Rev.

[3] Behere RV, Muralidharan K, Benegal V (2009). Complementary and alternative medicine in the treatment of substance use disorders--a review of the evidence. Drug Alcohol Rev..

[4] Hai AH, Franklin C, Park S, DiNitto DM, Aurelio N (2019). The efficacy of spiritual/religious interventions for substance use problems: a systematic review and meta-analysis of randomized controlled trials. Drug Alcohol Depend.

[5] Jilek-Aall L, Jilek WG, Pichot (1985). Buddhist temple treatment of narcotic addiction and neurotic-psychosomatic disorders in Thailand. Psychiatry: The state of the art (pp. 673–677).

[6] Sangkapreecha P, Sangkapreecha T (2013). The cave of healing: the physical/spiritual detoxification and the distinctive healing program for drug rehabilitation at Thamkrabok monastery, Thailand. Silpakorn Univ J Soc Sci Hum Arts.

[7] Peltzer K (1985). The therapy of alcoholics and cannabis smokers in the Zion Christian church in Malawi. Transcult Psychiatric Res Rev..

[8] Morojele N (1997). Indigenous approaches to the treatment of substance abuse in South Africa.

[9] Adthasit R, Kulsomboon S, Chantraket R, Suntananukan S, Jirasatienpong P, Petrakard P, Chantraket R (2007). The situation of knowledge management and research in the area of local wisdom in health care. The report situations of Thai traditional medicine, indigenous medicine and alternative medicine 2005–2007.

[10] Chan-iam W, Yodmalee B, Nakornriab M (2019). Thai traditional medicine at Wat nong Ya Nang Buddhist. Uthai Thani Province JFHB.

[11] Kaewla W, Wiwanitkit V (2015). Local primary health care by local religious center: a case study of a Mahayana Buddhist temple, Thailand. Ann Trop Med Public Health.

[12] Pengpid S, Peltzer K. Quality of life among patients with common mental disorders attending monk healers and primary care clinics in Thailand. JPMH. 2020. 10.1108/JPMH-01-2020-0003.

[13] Mbwayo AW, Ndetei DM, Mutiso V, Khasakhala LI (2013). Traditional healers and provision of mental health services in cosmopolitan informal settlements in Nairobi, Kenya. Afr J Psychiatry.

[14] Sorsdahl K, Stein DJ, Grimsrud A, Seedat S, Flisher AJ, Williams DR, Myer L (2009). Traditional healers in the treatment of common mental disorders in South Africa. J Nerv Ment Dis.

[15] Abbo C, Ekblad S, Waako P, Okello E, Muhwezi W, Musisi S (2008). Psychological distress and associated factors among the attendees of traditional healing practices in Jinja and Iganga districts, eastern Uganda: a cross-sectional study. Int J Ment Health Syst.

[16] Ngoma MC, Prince M, Mann A (2003). Common mental disorders among those attending primary health clinics and traditional healers in urban Tanzania. Br J Psychiatry.

[17] Musyimi CW, Mutiso VN, Musau AM, Matoke LK, Ndetei DM (2017). Prevalence and determinants of depression among patients under the care of traditional health practitioners in a Kenyan setting: policy implications. Transcult Psychiatry.

[18] Sooksompong S, Kwansanit P, Supanya S, Chutha W, Kittirattanapaiboon P, Udomittipong D (2016). The Thai National Mental Health Survey 2013: prevalence of mental disorders in megacities: Bangkok. J Psychiatr Assoc.

[19] Assanangkornchai S, Balthip Q, Edwards JG, assistance of the ASSIST-SBI development co-investigators (2014). Implementing the alcohol, smoking, substance involvement screening test and linked brief intervention service in primary care in Thailand. J Public Health.

[20] Pengpid S, Peltzer K, Puckpinyo A, Thammaaphiphol K (2016). Conjoint moderate or high-risk alcohol and tobacco use among male out-patients in Thailand. S Afr J Psychiatry.

[21] Serrano-Blanco A, Palao DJ, Luciano JV, Pinto-Meza A, Luján L (2010). Prevalence of mental disorders in primary care: results from the diagnosis and treatment of mental disorders in primary care study (DASMAP). Soc Psychiatry Psychiatr Epidemiol.

[22] John WS, Zhu H, Mannelli P, Schwartz RP, Subramaniam GA, Wu LT (2018). Prevalence patterns, and correlates of multiple substance use disorders among adult primary care patients. Drug Alcohol Depend.

[23] Luitel NP, Baron EC, Kohrt BA, Komproe IH, Jordans MJD (2018). Prevalence and correlates of depression and alcohol use disorder among adults attending primary health care services in Nepal: a cross sectional study. BMC Health Serv Res.

[24] Lee JD, Delbanco B, Wu E, Gourevitch MN (2011). Substance use prevalence and screening instrument comparisons in urban primary care. Subst Abus.

[25] Ward CL, Mertens JR, Flisher AJ, Bresick GF, Sterling SA, Little F (2008). Prevalence and correlates of substance use among south African primary care clinic patients. Subst Use Misuse.

[26] Prior K, Mills K, Ross J, Teesson M (2017). Substance use disorders comorbid with mood and anxiety disorders in the Australian general population. Drug Alcohol Rev.

[27] Wu LT, Zhu H, Ghitza UE (2018). Multicomorbidity of chronic diseases and substance use disorders and their association with hospitalization: results from electronic health records data. Drug Alcohol Depend.

[28] Ali R, Meena S, Eastwood B, Richards I, Marsden J (2013). Ultra-rapid screening for substance-use disorders: the alcohol, smoking and substance involvement screening test (ASSIST-lite). Drug Alcohol Depend.

[29] Kroenke K, Spitzer RL, Williams JB (2001). The PHQ-9: validity of a brief depression severity measure. J Gen Intern Med.

[30] Lotrakul M, Sumrithe S, Saipanish R (2008). Reliability and validity of the Thai version of the PHQ-9. BMC Psychiatry.

[31] Spitzer RL, Kroenke K, Williams JB, Lowe B (2006). A brief measure for assessing generalized anxiety disorder: the GAD-7. Arch Intern Med.

[32] Kocalevent RD, Berg L, Beutel ME, Hinz A, Zenger M, Härter M (2018). Social support in the general population: standardization of the Oslo social support scale (OSSS-3). BMC Psychol.

[33] Peltzer K, Pengpid S, Puckpinyo A, Yi S, Anh LV (2016). The utilization of traditional, alternative and complementary medicine for non-communicable diseases and mental disorders in health care patients in Cambodia, Thailand and Vietnam. BMC Complement Altern Med.

[34] Lee GB, Charn TC, Chew ZH, Ng TP (2004). Complementary and alternative medicine use in patients with chronic diseases in primary care is associated with perceived quality of care and cultural beliefs. Fam Pract.

[35] Peltzer K, Pengpid S (2019). The use of herbal medicines among chronic disease patients in Thailand: a cross-sectional survey. J Multidiscip Healthc.

[36] Rapier R, McKernan S, Stauffer CS (2019). An inverse relationship between perceived social support and substance use frequency in socially stigmatized populations. Addict Behav Rep.

[37] Lev-Ran S, Le Strat Y, Imtiaz S, Rehm J, Le Foll B (2013). Gender differences in the prevalence of substance use disorders among individuals with lifetime exposure to substances: results from a large representative sample. Am J Addict.

[38] Getachew T, Defar A, Teklie H, Gonfa G, Bekele A, Bekele A (2017). Magnitude and predictors of excessive alcohol use in Ethiopia: findings from the 2015 national non-communicable diseases STEPS survey. Ethiop J Health Dev.

[39] Kabwama SN, Ndyanabangi S, Mutungi G, Wesonga R, Bahendeka SK, Guwatudde D (2016). Alcohol use among adults in Uganda: findings from the countrywide non-communicable diseases risk factor cross-sectional survey. Glob Health Action.

[40] Center for Behavioral Health Statistics and Quality (2015). Results from the 2014 National Survey on Drug Use and Health: Detailed tables.

[41] Institute of Alcohol Studies. Older people’s drinking habits, 2017. Available at http://www.ias.org.uk/Alcohol-knowledge-centre/Alcohol-and-older-people/Factsheets/Older-peoples-drinking-habits-Very-little-very-often.aspx (Accessed 30 August 2020).

[42] Charitonidi E, Studer J, Gaume J, Gmel G, Daeppen JB, Bertholet N (2016). Socioeconomic status and substance use among Swiss young men: a population-based cross-sectional study. BMC Public Health.

[43] Tangcharoensathien V, Witthayapipopsakul W, Panichkriangkrai W, Patcharanarumol W, Mills A (2018). Health systems development in Thailand: a solid platform for successful implementation of universal health coverage. Lancet..

